# Orthopedic meta-implants

**DOI:** 10.1063/5.0179908

**Published:** 2024-01-19

**Authors:** Mohammad J. Mirzaali, Amir A. Zadpoor

**Affiliations:** Department of Biomechanical Engineering, Faculty of Mechanical, Maritime, and Materials Engineering, Delft University of Technology (TU Delft), Delft 2628CD, The Netherlands

## Abstract

Meta-biomaterials, engineered materials with distinctive combinations of mechanical, physical, and biological properties stemming from their micro-architecture, have emerged as a promising domain within biomedical engineering. Correspondingly, meta-implants, which serve as the device counterparts of meta-biomaterials, offer exceptional functionalities, holding great potential for addressing complex skeletal diseases. This paper presents a comprehensive overview of the various types of meta-implants, including hybrid, shape-morphing, metallic clay, and deployable meta-implants, highlighting their unprecedented properties and recent achievement in the field. This paper also delves into the potential future developments of meta-implants, addressing the exploration of multi-functionalities in meta-biomaterials and their applications in diverse biomedical fields.

## I. INTRODUCTION

Metamaterials and meta-devices have their roots in optics^1,2^ and electromagnetics,^3–5^ where designer-architected arrays of (micro-) devices at a smaller scale are used to create materials and devices at a larger scale that have unusual or unprecedented properties and functionalities. An important example of such types of metamaterials are invisibility cloaks.^6–8^ Since the 2010s, there has been growing interest in other types of metamaterials and meta-devices with unusual mechanical,^9–14^ acoustic,^15,16^ thermal,^17,18^ or bio-inspired^19–21^ properties and functionalities. The type of the targeted property is usually mentioned in the terminology used to refer to these types of metamaterials. Examples are mechanical metamaterials,^22–24^ acoustic metamaterials,^25^ thermal metamaterials,^26,27^ and meta-biomaterials.^28–31^ The primary focus of the current article is the biomedical applications of metamaterials, which is why we will be limiting the discussion to meta-biomaterials.

Meta-biomaterials are engineered, architected materials with unusual, rare, or unprecedented combinations of mechanical, physical (e.g., mass transport), and biological properties.^28^ Similar to other types of metamaterials, the exotic properties of meta-biomaterials result from their micro-architecture. The definition of micro-architecture is quite broad and includes both geometrical design at the macro-, micro-, and nanoscales as well as the spatial distribution of various types of materials within the structure of meta-biomaterials. However, the focus of most developments reported to date has been the geometrical design of meta-biomaterials.^32–38^

Meta-implants are the device-type equivalent of “meta-biomaterials.” Meta-implants are implantable medical devices that offer rare or unprecedented functionalities.^39–41^ Meta-implants may benefit from the exotic properties of meta-biomaterials to create novel types of functionalities or create those functionalities through a more direct route. In this article, we will discuss the main types of meta-implants that have appeared in the literature and will provide some perspectives regarding the possible future developments in this exciting area of research. While the concepts and methods discussed in this article are applicable to a wide range of implantable medical devices, we will primarily focus on orthopedic meta-implants.

## II. WHY META-IMPLANTS?

Primary orthopedic surgeries using joint-replacement implants are one of the most successful types of treatments, providing pain relief, mobility, and improved quality of life for 10–20 years with implant survival rates of >80% even after 15 years of implantation.^42^ In contrast, there are many other types of complex skeletal diseases for which orthopedic implants are highly underperforming. Examples include revision surgeries,^43–45^ tumor resections,^46–49^ trauma surgeries,^50,51^ and other types of complex bony reconstructions.^52,53^ The implants used for such types of procedures often fail prematurely due to aseptic loosening^54–56^ or implant-associated infections,^57–59^ among other reasons. Patient satisfaction rates are also quite low.^46,60,61^ To put this in perspective, the survival rate of the implants used after tumor resections can be <20%^62–65^ after 5 years, as compared to >85% survival rate of primary hip implants.^42^ As for implant-associated infections, rates of 15%–50% are not uncommon in complex bony reconstructions,^66^ as compared to 1%–9% in primarily orthopedic surgeries.^67^ The primary aim in the development of orthopedic meta-implants is to address the challenges associated with the treatment of complex bony diseases, particularly (1) improving the longevity of the implants used for such procedures through enhanced primary and secondary fixations and (2) decreasing the rate of implant-associated infections.

Meta-biomaterials are generally developed to achieve one or both of the above-mentioned aims by offering unusual combinations of properties that are otherwise extremely difficult or impossible to achieve.^68–70^ These unusual combinations of properties must be eventually used in an implantable medical device for application in clinical settings. This is achieved using the meta-implants that are described in this paper. However, using the unusual properties of meta-biomaterials is not the only way for meta-implants to improve the treatment of complex bony diseases. As we will later see, it is possible to create the unusual functionalities at the device-level. Of the four following sections, the first two are primarily focused on the use of the unusual properties of meta-biomaterials for the improvement of the functionality of meta-implants, while the last two concern the direct development of functionalities.

## III. HYBRID AUXETIC-CONVENTIONAL META-IMPLANTS

**FIG. 1.**
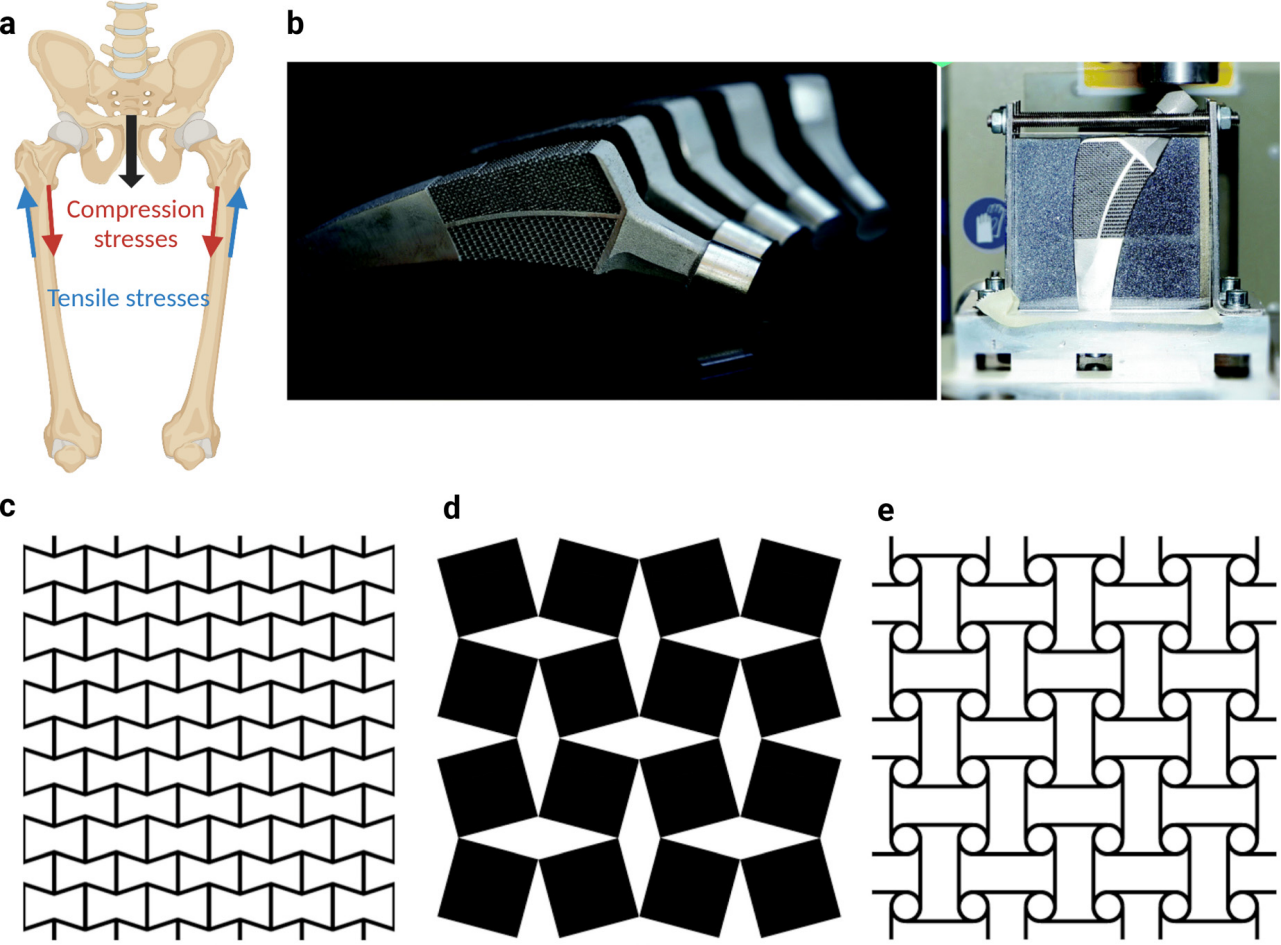
Under physiological conditions, the lateral side of the hip stem may undergo tensile deformation while compressive loading affect the medial side (a). The SLM 3D-printed hybrid meta-implant prototype, fabricated from Ti-6Al-4V, features a combination of auxetic and non-auxetic micro-architectures (b).^68^ Kolken *et al.*, Mater. Horiz. **5**(1), 28 (2018). Copyright 2018 Author(s), licensed under a Creative Commons Attribution (CC BY License). Examples of auxetic unit cells, including reentrant (c), rotating rectangles (d), and anti-chiral (e) structures. Subfigures (c)–(e) are reprinted with permission from Borovinšek *et al.*, Mater. Sci. Eng.: A **795**, 139914 (2020). Copyright 2020 Elsevier.^146^

Natural materials, save for a limited number of exceptions, exhibit a positive Poisson's ratio, meaning that they contract orthogonally to the direction that experiences normal stresses (e.g., a cylinder loaded along its length contracts along its diameter). The metallic biomaterials commonly used to manufacture orthopedic implants all exhibit a positive Poisson's ratio. In some cases, such as a hip stem, this could be suboptimal. Hip stems are mechanically loaded under bending. In bending, one side of the bent beam (i.e., the hip stem) experiences tension while the other side experiences compression [Fig. 1(a)]. For example, the lateral side might be experiencing tension while the medial side is under compression. For materials with a positive Poisson's ratio, the side that is under compression becomes thicker, thereby moving toward the bone at the implant–bone interface.^68^ This improves the bone–implant contact because the cyclic loading of the hip joint during gait continually loads the bone under compression, stimulating bony ingrowth. Moreover, it will be more difficult for the wear particles detached from the implant's articulating surface to lodge in-between the implant and the bone and create the inflammatory processes that may lead to aseptic loosening.^71–73^ On the side of the implant that is under tension, however, the opposites of all these phenomena occur as the implant contracts in the medial–lateral direction, moving away from the bone. This tensile load may cause fracture at the bone–implant interface, which would allow the wear particle to lodge in-between the bone and implant and give rise to an inflammatory process.

Hybrid auxetic-conventional meta-implants [Fig. 1(b)] can address this challenge by creating architected (metallic) meta-biomaterials with a negative Poisson's ratio.^34,68^ Such materials are referred to as auxetic materials and can be created in different ways through various types of micro-architectural designs [Figs. 1(c) and 1(e)]. Given that an auxetic material becomes thicker under tension, positioning an auxetic meta-biomaterial on the side of the hip stem experiencing tension could prevent the implant from moving away from the bone, thereby limiting the above-mentioned adverse effects. It is important to realize that using an auxetic material throughout the body of the implant will not solve these challenges we mentioned before, as it will simply move the potential adverse effects from the side experiencing tension to the one experiencing compression. Combining an auxetic meta-biomaterial with another meta-biomaterials with a positive Poisson's ratio (i.e., a conventional meta-biomaterial) could address this challenge. In such a scenario, the meta-biomaterial with a positive Poisson's ratio will be positioned on the side of the implant experiencing compression while the auxetic meta-biomaterial will be on the side experiencing tension.

**FIG. 2.**
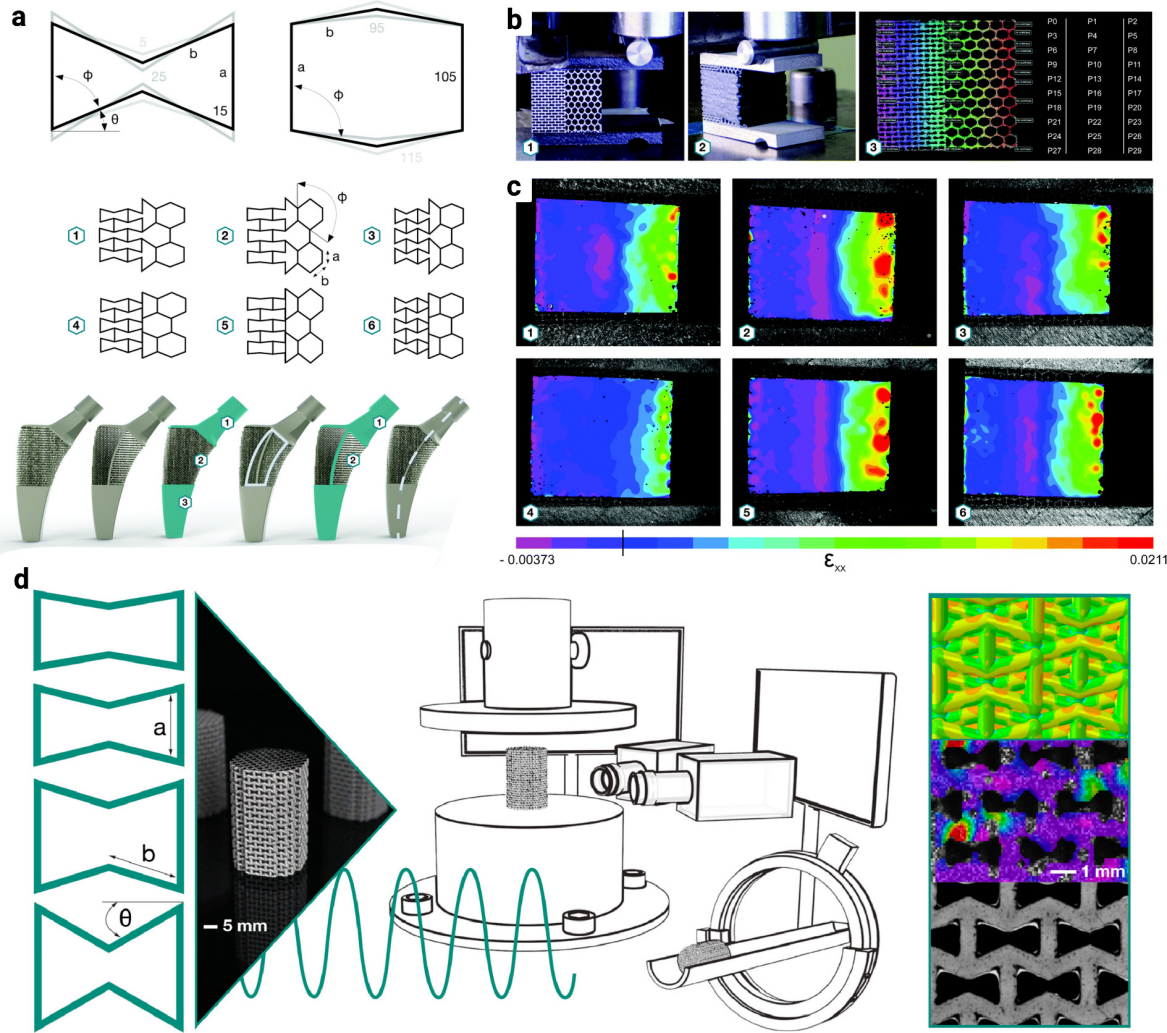
Some examples of hybrid meta-implants. (a) The design procedure for integrating auxetic and conventional structures to create hybrid meta-implants. Some examples of hybrid meta-biomaterial designs with varied internal unit cell angles. The experimental setup to conduct compression tests (b), and digital image correlation results visualizing the strain distribution in those meta-biomaterial lattice structures (c). Subfigures (a)–(c) are reproduced with permission from Kolken *et al.*, Mater. Horiz. **5**(1), 28 (2018). Copyright 2018 Author(s), licensed under a Creative Commons Attribution (CC BY License).^68^ An array of characterization methodologies, including cyclic fatigue testing, DIC, and computational modeling, can be used to evaluate the initiation and propagation of fatigue cracks in auxetic meta-biomaterials (d).^74^ Kolken *et al.*, Acta Biomater. **138**, 398–409 (2022). Copyright 2022 Author(s), licensed under a Creative Commons Attribution (CC BY License).

A prototype of such a meta-implant was developed and additively manufactured from Ti-6Al-4V in a recent study [Fig. 1(b)].^68^ The hybrid hip stem was made by joining an auxetic porous biomaterial designed on the basis of reentrant unit cells to a conventional porous biomaterials designed using a honeycomb unit cell. The two sides of the implant were joined using a solid part that provided additional mechanical properties and enabled the transition from one type of the unit cell to the other. Six versions of these meta-implants were designed and 3D printed [Fig. 2(a)]. They were then tested in a simulated-implantation experiment in which the implants were positioned in a bone-mimicking phantom and were subjected to compressive loading. The full-field strain patterns were measured using digital image correlation (DIC). The results of these measurements confirmed the presence of compression on both sides of hybrid implants combining auxetic and conventional meta-biomaterials while corresponding implants using either positive or negative values of the Poisson's ratio created tension on one of their sides and compression on the other side [Fig. 2(b)].

Considering the geometrical design of hybrid meta-implants, the reentrant hexagonal honeycomb unit cell is often the design of choice, primarily due to the simplicity inherent in the creation of reentrant structures. The efficiency of such (non)auxetic meta-biomaterials relies heavily on such variables as the reentrant angle, aspect ratio, and relative density of the unit cell in the meta-biomaterials. Despite its bending-dominated design, the reentrant hexagonal honeycomb mirrors the stress resilience of certain stretch-dominated unit cells, showcasing an exceptional fatigue performance with an average maximum design stress of 0.47 $\sigma_{y}$ at 10^6^ cycles (range: 0.35 $\sigma_{y}$–0.82 $\sigma_{y}$) for auxetic structures fabricated via the selective laser melting (SLM) technique from commercial pure titanium (CP-Ti).^34^

Another fundamental consideration in the design of hybrid meta-implants is understanding crack propagation in these materials under cyclic loading conditions, particularly at the interface of auxetic-non-auxetic structures. Given that these materials may undergo repeated stresses in clinical applications, understanding how they fail is of paramount importance. A recent study performed compression–compression fatigue tests on 12 different meta-biomaterial designs, documenting the full-field strain measurement via DIC.^74^ The fatigue tests were intermittently halted to allow for the inspection of micro-architectural damage in the specimens using micro-computed tomography (*μ*CT)^74^ [Fig. 2(c)]. Such microscale analyses are essential as they can reveal accumulated damage that might be invisible at the macroscopic level. Microscale damage, in fact, can potentially jeopardize the success of a meta-implant. For instance, the release of powder particles from damaged lattices could trigger a foreign body response in the patient, leading to adverse outcomes. Therefore, the determination of a suitable failure criterion necessitates a comprehensive approach, taking into account both macroscopic and microscopic assessments.

The clinical application of auxetic meta-biomaterials presents numerous challenges. One particular hurdle is the enhancement of the mechanical properties of meta-biomaterials by increasing its relative density, a process which could inadvertently lead to reduced porosity, smaller pore size, and lower permeability. However, the strategic use of minimal surface patches, carefully designed to increase surface area, can address this issue without substantially impacting the mechanical properties.^75^

## IV. SHAPE-MORPHING AND SHAPE-SHIFTING META-IMPLANTS

Distinct from patient-specific 3D printed implants (PSIs), which although beneficial are labor-intensive, time-consuming, and costly due to the patient-specific design process, shape-morphing meta-implants offer a generic, cost-effective, and readily alternative with equivalent customization potential. The emerging field of shape-morphing meta-implants teems with innovation, from the use of non-auxetic meta-biomaterials to the deployment of self-folding techniques^28,76–79^ and automated folding methods.^80^ These new-generation shape-morphing meta-implants hold the promise of overcoming the shortcomings of the currently available patient-specific implants, potentially transforming the orthopedic implant landscape.

**FIG. 3.**
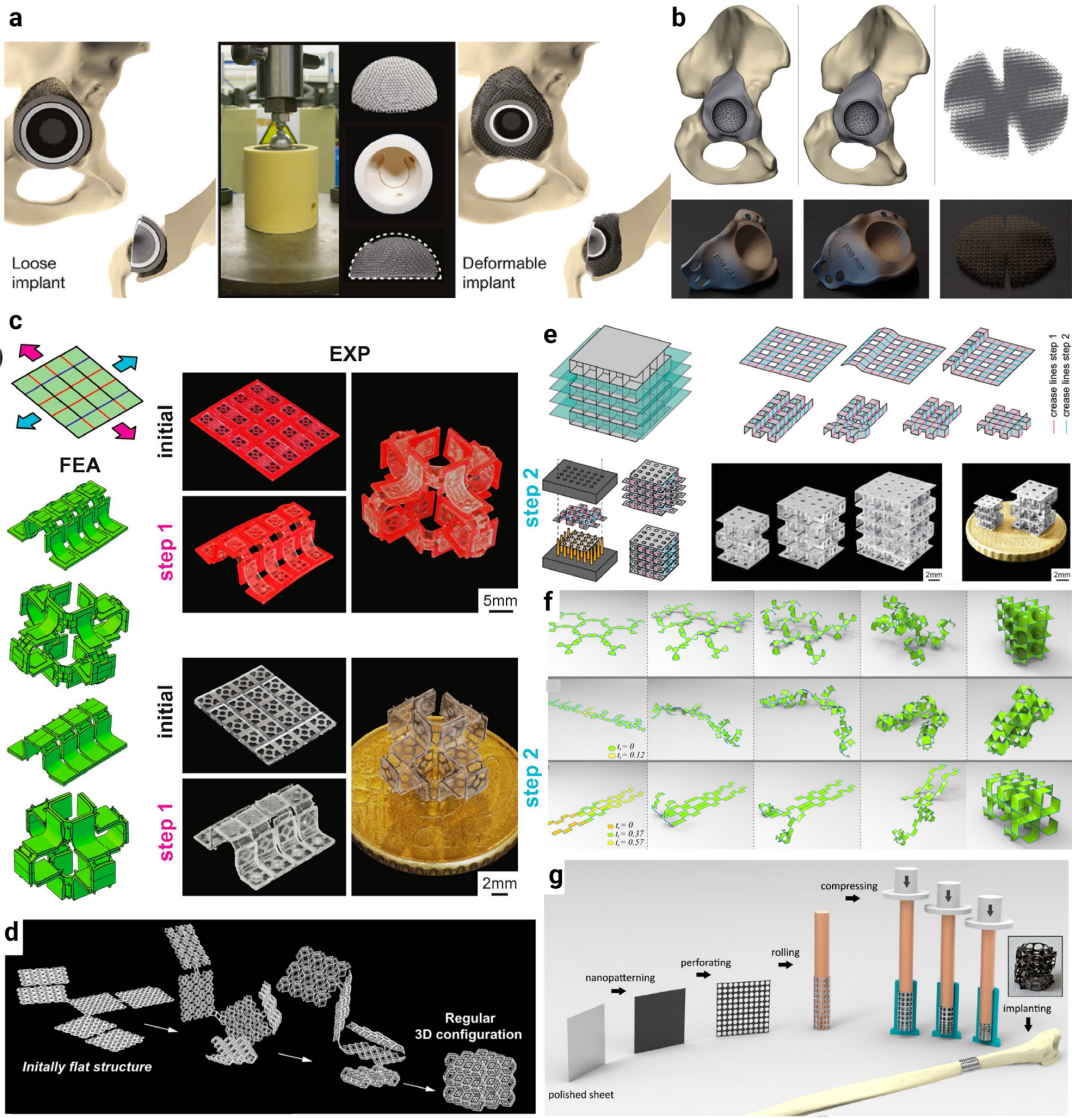
Some examples of shape-morphing meta-implants. (a) An example of a deformable meta-implant, made from non-auxetic unit cells, suitable for filling spatial voids in acetabular defects.^82^ Kolken *et al.*, Acta Biomater. **125**, 345–357 (2021). Copyright 2021 Author(s), licensed under a Creative Commons Attribution (CC BY License). (b) Some examples of triflange design (top) and manufactured acetabular cups (bottom) accompanied by a separate deformable titanium mesh.^83^ Magré *et al.*, 3D Print. Med. **9**(1), 16 (2023). Copyright 2023 Author(s), licensed under a Creative Commons Attribution (CC BY License). (c) A case of sequential shape transition from a flat state to a complex multi-story configuration.^77^ van Manen *et al.*, Mater. Today **32**, 59–67 (2020). Copyright 2020 Author(s), licensed under a Creative Commons Attribution (CC BY License). (d) An example of a self-folding origami lattice structure, demonstrating a transformation sequence from an initially flat structure to a 3D structure with regular configuration.^76^ Janbaz *et al.*, Sci. Adv. **3**(11), eaao1595 (2017). Copyright 2017 Author(s), licensed under a Creative Commons Attribution (CC BY License). (e) An example of automated two-step folding of origami lattices to create stiff meta-biomaterials. Reprinted with permission from van Manen *et al.*, Small **19**(3), 2203603 (2022). Copyright 2022 John Wiley and Sons.^80^ (f) Examples of folding periodic minimal surface meta-biomaterials through the assembly of multiple units.^90^ Callens *et al.*, Appl. Mater. Today **15**, 453–461 (2019). Copyright 2019 Author(s), licensed under a Creative Commons Attribution (CC BY License). (g) The use of a crumpling method to create 3D metallic meta-biomaterials from initially flat sheets.^89^ Ganjian *et al.*, Mater. Des. **220**, 110844 (2022). Copyright 2022 Author(s), licensed under a Creative Commons Attribution (CC BY License).

One pioneering design uses non-auxetic meta-biomaterials^81^ to create deformable porous outer layers for shape-morphing meta-implants. In such designs, the aim is to enhance the initial stability of the implant and stimulate the surrounding bone homogeneously for the treatment of acetabular bone defects, commonly encountered in the revision of total hip replacement (THR) surgeries.^82^ A prototype of such meta-implants, featuring a deformable porous outer layer, was additively manufactured using pure titanium lattices^82^ [Fig. 3(a)]. Among these shape-morphing meta-implants, lattices with a functionally graded diamond infill showed the most promising space-filling behavior. Despite this promise, the push-in forces required for implantation may exceed those of current surgical practices, indicating the need for optimization.

Relatively recently, an inventive strategy for acetabular revision surgery has been proposed, involving custom-made triflange acetabular shape-morphing meta-implants.^83^ This design introduces a novel concept for the triflange, incorporating a deformable porous titanium layer that redirects forces from the trabecular rim to the bone stock behind the implant, thereby mitigating further stress-shielding [Fig. 3(b)]. Two strategies were employed to achieve this: engineering a deformable layer at the back of the implant or adding a generic deformable mesh behind the implant. To assess their effectiveness, these implants were tested in sawbones with acetabular defects and subjected to a cyclic compression test of 1800 N for 1000 cycles. The results showed that primary implant fixation and stability could be achieved in simulated large acetabular revision surgery using a deformable titanium layer behind the cup. This represents a significant advancement in the application of these innovative materials and techniques in surgical procedures.

The design of shape-morphing meta-implants with either self-folding or automated folding methods requires a folding strategy to transform flat sheets into geometrically complex 3D cellular materials. The practical challenges of folding, particularly in traditional origami, necessitate the development of more tunable self-folding techniques, especially for complex structures at varying scales. The lattice kirigami technique^77,84,85^ provides a more applicable solution due to its simple, repetitive folding pattern and its ability to maintain intrinsic flatness, regardless of the folding configuration.

There are two main strategies for programming the shape-shifting behavior of flat (soft) matter into 3D shapes: bending and buckling.^86,87^ The bending strategy involves programming a stress gradient along the material thickness, manipulating geometrical parameters, material properties, and programming and activation conditions to achieve the desired 3D configuration. On the other hand, the buckling strategy, driven by instability, facilitates a wide range of shapes with specific target values of both mean and Gaussian curvatures. However, controlling the direction of out-of-plane deformation remains a challenge due to the nature of instability. Combining both strategies could potentially expand the range of achievable target shapes. Additionally, the concept of sequential folding,^77,78,86^ which involves planning the folding sequence to avoid self-collision between panels, contributes to extended shape possibilities and improved structural integrity.

The lattice kirigami technique with its relatively simple folding pattern is well-suited for self-folding applications and the transformation of flat sheets into curved geometries. It introduces mechanical self-folding, activated by global stretching, and is broadly applicable to various materials^77^ This technique enables the sequential self-folding of multi-story constructs, even at microscale dimensions [Fig. 3(c)].^77,80^ The resulting surface-based topologies demonstrate superior fatigue resistance and promote better bone growth as compared to traditional truss-based designs.^88^ The combination of high porosity and shape adaptability holds promise for facilitating implantation via minimally invasive surgery.

A specific application of kirigami-enabled self-folding origami includes the combination of two types of permanently deforming kirigami elements, operating based on multi-stability or plastic deformation, with an elastic layer to create self-folding basic elements [Fig. 3(c)].^77^ The folding angles of these elements can be controlled through kirigami cut patterns and elastic layer dimensions, accurately predicted using computational models. These basic elements are modularly assembled to form complex 3D structures, including multi-story origami lattices, with varying sizes and microscale feature sizes. Starting from a flat state enables the incorporation of precisely controlled, complex, and spatially varied micropatterns, as well as flexible electronics, into the self-folded 3D structures. This allows for the creation of multifunctional and instrumented implantable medical devices.^77^

An example of using an automated folding method to fabricate shape-shifting meta-biomaterials involves creating origami lattices from initially flat sheets [Fig. 3(d)].^76^ These sheets can be further functionalized for multi-functional properties, such as surface decoration with nantopographical ornaments. The ability to adjust the unit cell type enables the resulting meta-biomaterials to offer a broad spectrum of mechanical and physical properties as well as other functionalities.^80^ Specifically, a newly developed automated folding technique introduces sharp folds into thick metal sheets, enhancing their stiffness [Fig. 3(e)].^80^ The scalability of this technique is demonstrated by fabricating origami lattices with over 100 unit cells and unit cells as small as 1.25 mm, using laser micromachining. Assembling the folded stories involved the use of a biocompatible cyanoacrylate-based adhesive, ensuring sufficient fixation by subjecting the specimens to compression for at least 2 min. Prior to folding, the surfaces of the sheets were nanopatterned and protected by a thin coating layer that remained intact during the folding process.

The automated folded origami lattices successfully meet the primary design objectives for a meta-biomaterial, encompassing biocompatibility, bone-mimicking mechanical properties, osteogenic behavior, suitable pore sizes, scalability, and customizable surface nanopatterns. Notably, the nanopatterned folded specimens exhibit significantly increased mineralization compared to their non-patterned counterparts.^80^ This suggests the potential benefits of the proposed approach in the field of biomaterials, particularly in applications requiring the combination of complex 3D shapes with precise and controlled surface nanopatterns. However, it is important to consider the complexity, cost, long-term stability, and biocompatibility implications of the coating layer used to preserve nanoscale features during the folding process.^80,85,89^ Further investigation is, therefore, needed in these areas.

In addition to traditional origami techniques that result in originally flat 3D structures, a more advanced approach has been proposed to bridge the Euclidean nature of origami with the hyperbolic nature of triply periodic surfaces (TPMS), offering novel possibilities in the 2D-to-3D fabrication paradigm and the design of architected materials with enhanced functionality [Fig. 3(f)].^90^ This approach harnesses material stretching and kinematic joints to address non-developability of hyperbolic surfaces, capitalizing on the inherent hyperbolic symmetries of TPMS to assemble intricate 3D structures. The process involves attaching 3D-printed foldable frames to pre-strained elastomer sheets, which, upon the release of the pre-strain, enable self-folding and self-guided minimal surface shape adaptation.^90^ By facilitating the connection of multiple patches through vertex or edge connections, a wide variety of foldable 3D structures can be created.

Controlled crumpling [Fig. 3(g)] is another approach for fabricating shape-shifting meta-biomaterials.^89,91,92^ For instance, titanium (Ti) nanopatterns were created on polished Ti sheets using laser cutting and reactive ion etching (RIE), followed by crumpling at two different deformation velocities.^89^ The resulting specimens showed the desired geometrical and adjustable mechanical properties and demonstrated cytocompatibility *in vitro*.

## V. METALLIC CLAY

**FIG. 4.**
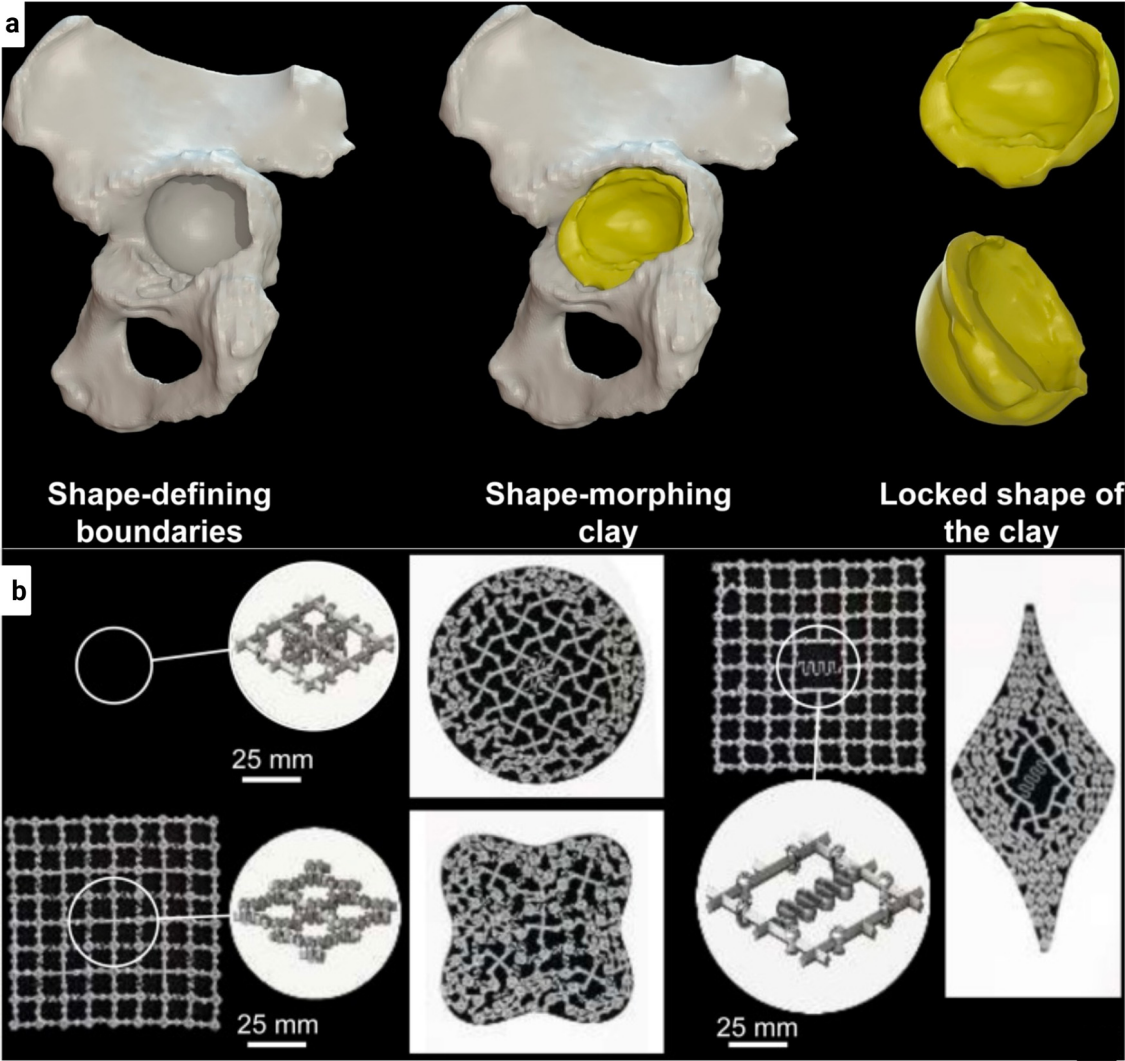
(a) The presented schematic drawing illustrates the fundamental concept of metallic clay. (b) Three prototypes of planar shape-morphing structures equipped with compliant locking mechanism. Reprinted with permission from Leeflang *et al.*, Addit. Manuf. **28**, 528–534, (2019). Copyright 2019 Elsevier.^93^

The concept of “metallic clay” meta-implants has been recently introduced.^93^ This uniquely engineered material can be molded into complex shapes and subsequently shape-fixed, mirroring the behavior of clay before and after firing. A distinctive characteristic of this material is its dual state, transitioning between a “universal shape morphing” state and a “locked-shape” state (Fig. 4).^93^ It is worth noting that the successful application of metallic clay demands a novel approach to joint and locking mechanism design to ensure compatibility with metal 3D printing techniques.

Multiple joints and locking mechanisms facilitate the simulation of these two distinct states in metallic clay. These joints provide localized degrees of freedom, allowing the material to be shaped into a diverse range of complex shapes. Once the desired shape is achieved, a locking mechanism is activated to stabilize the structure, a process analogous to the firing of clay.

The significance of this material lies in its potential to serve as a key component in the development of “combined shape-morphing and shape-locking meta-implants.” These implants hold promise to provide cost-effective, universally applicable alternatives to PSIs. Although PSIs have their advantages, they are burdened by laborious, time-consuming, and expensive customization processes. The versatile nature of metallic clay offers a novel approach to customizing implant shapes, thereby circumventing these limitations.

However, the intricate process of 3D printing self-supporting, miniaturized, non-assembly joints, combined with equally small locking mechanisms using metals, presents considerable challenges. Overcoming these challenges require a series of design innovations, particularly in the realm of joints and locking mechanisms. Moreover, the scalability of these innovations and their reproducibility in a manufacturing setting remain areas of ongoing investigation.

Complementing this folding technique is the application of a multibody kinematic system approach^94,95^ in the design of shape-morphing meta-biomaterials. This approach introduces the concepts of “nodes,” “links,” and “bodies” to delineate the components of the metamaterial. Within the structure, kinematic pairs serve as joints, enabling specific types of motion between nodes.^94^ These shape-morphing metamaterials have the potential to create complex structures with dynamic properties. Nevertheless, more research is required to address challenges associated with singularity issues resulting from distance constraints and to facilitate the creation of larger structures.

## VI. DEPLOYABLE META-IMPLANTS

Deployable meta-implants have recently emerged as revolutionary devices with diverse applications. Their compact designs permit implantation via minimally invasive surgical procedures, which necessitate the application of an external force. This method notably diminishes the invasiveness of surgical interventions (e.g., kyphoplasty), facilitating quicker recovery periods and lowering the probability of post-operative complications. The main advantages of these devices stem from the small incisions needed for their insertion into the body.^96^

**FIG. 5.**
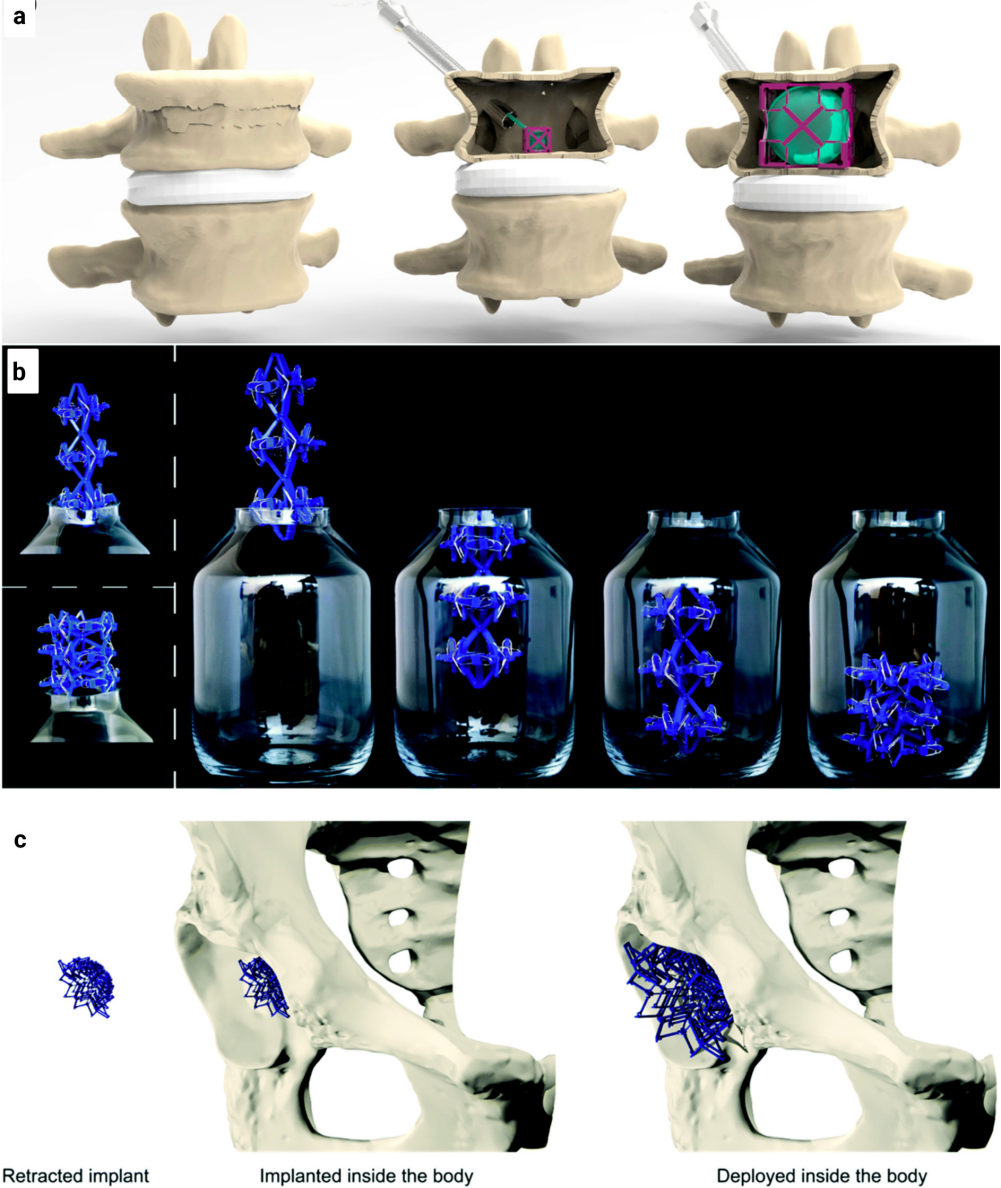
Some examples of deployable meta-implants. (a) The innovative concept of deployable implant for addressing vertebral compression fractures. Using a minimally invasive surgical approach, a deployable structure, incorporating a balloon, can be inserted within the fractured vertebra. The balloon inflation subsequently triggers the expansion of the deployable structure, restoring the original height of the vertebral.^97^ Bobbert *et al.*, Mater. Des. **191**, 108624 (2020). Copyright 2020 Author(s), licensed under a Creative Commons Attribution (CC BY License). (b) An example of how a multi-stable structure can be inserted into a bottle, even when the deployed structure does not accommodate the opening. (c) A potential application of multi-stable structures as bone implants.^102^ Bobbert *et al.*, J. Mater. Chem. B **6**(21), 3449–3455 (2018). Copyright 2018 Author(s), licensed under a Creative Commons Attribution (CC BY License).

A novel concept of deployable implants has been proposed for the treatment of vertebral compression fractures. This innovative idea merges the principles of origami, kirigami, and multi-stability to engineer a new generation of deployable meta-implants.^97^ These devices are inspired by the multi-layered design characteristics of Russian dolls and natural structures [Fig. 5(a)].^97^ They utilize design strategies that allow systematic adjustments of the deployment force, deployment ratio, mechanical properties, pore size, and porosity of meta-implants. The bio-functionality of these deployable meta-implants is also dependent on the design of kirigami cut patterns, the thickness of the metal sheet, and the number of layers in the Russian doll designs. Furthermore, the potential of surface nanopatterns to direct stem cell differentiation and mitigate implant-associated infections has been recognized.^98–101^

The methodology applied to deployable meta-implants for the treatment of vertebral compression fractures deserves special attention due to its ability to adjust the mechanical properties of the meta-implant and its dimensions significantly.^97^ Each additional layer leads to a multi-fold enhancement in the force corresponding to the same displacement, thereby reinforcing the implant for scenarios necessitating higher forces. Techniques typically employed on flat surfaces, such as electron beam lithography, reactive ion etching (RIE), and electron beam induced deposition (EBID), could be used to simultaneously stimulate the osteogenic differentiation of stem cells, and eliminate bacteria. Various types of surface micropatterns and nanopatterns can be engraved onto the flat sheet specimen, which can then be folded and deployed as meta-implants with different surface-related functionalities.^97^ The incorporation of high porosity and surface nanopatterns could stimulate bone regeneration and, thus, secondary fixation of the meta-implants.

The development of two types of basic bi-stable elements with single curved and doubly curved side hinges provides another methodology for creating deployable meta-implants.^102^ These elements consist of flexible components acting as joints and rigid components fulfilling structural functions. By connecting these basic bi-stable elements, more complex (multi-stable) mechanisms can be accomplished. This process enables the creation of multi-stable structures with differing deployment and retraction behaviors, presenting potential applications as bone implants^102^ [Figs. 5(b) and 5(c)].

Despite their transformative potential, these deployable meta-implants do have some limitations when manufactured using the selective laser melting (SLM) process. The primary challenge is that the process often grapples with accuracy issues during printing. Furthermore, it has been found that brittle fracture is more common in the as-built structures, highlighting the need for further research to improve the microstructure of as-built SLM specimens to increase their ductility.^96^ Moreover, many aspects of deployable meta-implants, including their behavior under dynamic loading conditions, require further investigation before their integration into clinical practice.

## VII. DISCUSSION AND FUTURE DIRECTIONS

Meta-biomaterials offer a plethora of opportunities for the design of meta-implants, each with their unique design requirements contingent upon the application. Critical to the design process is the adoption of a rational (micro-)architecture design, informed by physical theories and computational models, which typically involves computationally intensive simulations. Recent advancements have demonstrated the potential of machine learning techniques, including physics-informed artificial intelligence (AI) tools in general and deep learning in particular, for addressing inverse design problems, thereby aiding in the retrospective calculation of meta-implant micro-architectures.^103,104^ It is, however, crucial to address potential challenges, such as overfitting and substantial data requirements when applying these techniques.

To exercise independent control over the mechanical and mass transport properties of meta-biomaterials, the employment of parametric design strategies can be beneficial. This could be realized through a combination of various types of lattice structures (e.g., strut- and surface-based^75,105^) alongside random-based designs^106–109^ and the integration of bio-inspired design paradigms (e.g., functional gradients^110–114^). Such strategies aid in alleviating stress concentrations at the bone–implant interface and in locally tuning properties for enhanced bone tissue integration. A burgeoning field of research involves the use of multi-objective numerical optimization methodologies (e.g., gradient-based^115^) in constructing 3D micro-architected meta-implants. The primary objective of such methodologies should be to mitigate interface fracture risk, optimize implant-induced bone remodeling, and reduce the risk of implant failure, by leveraging multi-physics simulations that account for such factors as the spatial distribution of mechanical properties (e.g., auxetic vs non-auxetic properties), the effects of cyclic loading effects on the biodegradation rate of biodegradable implants,^116,117^ and the crack initiation in meta-implant designs.^118,119^

In the design of multi-functional meta-implants, some additional factors such as material choice, surface bio-functionalization, the effects of tissue ingrowth on the mechanical properties of the implant–bone complex,^120^ and drug delivery require further consideration.

As far as the selection of materials is concerned, a wide range of materials from metals and their alloys to, polymers, ceramics, and different composites can be used for adding additional functionalities to meta-biomaterials in general and meta-implants in particular. The type of the 3D printing fabrication technique dictates the choice of materials, as some 3D printing techniques are only or primarily compatible with specific materials. Here, we chiefly focus on metals and their alloys, particularly those fabricated using powder bed fusion processes, including SLM. More information regarding the different types of polymers and/or ceramics can be found elsewhere.^36,121^ The focus on metallic biomaterials is motivated by the fact that orthopedic meta-implants, particularly those used for load-bearing applications, require much higher mechanical properties, including strength and toughness, than the one offered by polymeric and ceramic materials, respectively.

Several examples of metallic meta-biomaterials demonstrate how the choice of material can add another level of functionality to the biomaterial. For instance, specific types of metals and their alloys can provide a higher corrosion resistance properties as well as a high biocompatibility (e.g., Tantalum^122^), a high fatigue resistance (e.g., CoCr alloys^123,124^), a high ductility and good biocompatibility (e.g., pure titanium^125,126^), controlled biodegradability [e.g., magnesium (WE43)^127^], or superelasticity and shape memory properties (e.g., NiTi alloys^128^). These properties are beneficial for creating advanced medical devices with multiple functionalities. *In situ* alloying is another process for mixing several materials to achieve customized properties and functionalities.^129^ One of the main aims of using *in situ* alloying is to introduce ceramic reinforcing particles into a metallic matrix to form a composite material that combines the mechanical properties of the metallic phase with the biological functionalities of the ceramic phase.^130^

Other additive manufacturing (AM) techniques, such as inkjet or extrusion-based printing,^131^ have also been used to expand the range of the materials of use. Examples include metals with biodegradable properties, such as magnesium,^116,132,133^ Zn-alloys,^134^ and iron-based.^127,135,136^ These pre-alloyed powders can be mixed with a binder polymeric system in an ink to create a homogeneous dispersion. The resulting scaffold is then finalized in a furnace after debinding and sintering. Biodegradable meta-implants have shown promising fatigue resistance in the presence of the simulated body fluid than in air.^117^ Finally, renewable and recycled waste materials represent a novel source of raw materials for biomaterials and tissue engineering applications, particularly for sustainable development.^137^

The advent of multi-material AM offers great opportunities for the spatial distribution of multiple materials within the meta-implant design. Of the various AM techniques, extrusion-based 3D printing has shown promise in fabricating biodegradable iron-manganese scaffolds, which are non-ferromagnetic and demonstrate enhanced biodegradation rates.^138^ These scaffolds also exhibit weakly paramagnetic behavior, making them suitable as MRI-compatible bone substitutes, with *in vitro* biodegradation rates aligning with the ideal range for bone substitution.^138^

Optimization of ink formulations for printability and adjusting other bio-ceramic powder particles, such as β-tricalcium phosphate (TCP),^134^ can facilitate the creation of multi-functional bone substitute meta-biomaterials with modulated scaffold biodegradation rates. Biofunctionalized porous meta-scaffolds embedded with silver and Fe nanoparticles can also be present as a strategy to circumvent bacterial resistance to inorganic nanoparticles and antibacterial coatings.^139,140^ Such coatings are designed to prevent implant-associated infections and promote bone tissue regeneration. Surface biofunctionalization of meta-implants with complex geometries can be performed with the help of plasma electrolytic oxidation (PEO).^141^ Such meta-biomaterials have exhibited strong antibacterial behavior and improved osteogenic differentiation of mesenchymal stem cells.^142^

An emerging area of exploration is the application of 4D printing techniques in fabricating shape-morphing meta-implants, entailing the discovery of novel designs and models to predict the bending behavior and stiffness of elements.^143^ 4D printing facilitates the creation of meta-implants with dynamic properties. Despite the inherent challenges in creating multiscale meta-biomaterials, their programmable shape deformation holds significant promise. There remains, however, room for further exploration in devising effective folding strategies for complex structures and in surmounting the geometrical barrier when folding minimal surfaces from a flat state.

Some of the biological and pre-clinical aspects of the meta-biomaterials have been investigated using *in vivo* animal models. An example includes understanding the effect of antibacterial coatings applied to AM meta-biomaterials on implant-associated infection and the associated immune response coating.^144,145^ However, the research on osteoimmunomodulation is still in progress. A comprehensive understanding of the biological response of meta-biomaterials, along with their long-term mechanical and biological properties needs further elucidation and investigation. In this context, the implementation of auxetic meta-biomaterials poses challenges, such as isolating the effects of the Poisson's ratio from other properties, conducting research with diverse cell types, and performing *in vivo* experiments.^70^ The biological response to these materials remains unclear, and future research should focus on understanding their impact on bone cells and implant performance under different loading regimes.

## VIII. IMPLICATIONS AND PERSPECTIVE

The emergence of meta-biomaterials and meta-implants, as discussed in the preceding sections, signifies a paradigm shift in biomedical engineering. Existing research highlights the technical merits of these meta-implants, such as enhanced structural features and the incorporation of advanced 4D printing techniques. Nevertheless, the broader implications of this technology are often insufficiently discussed. These novel implants offer opportunities for more personalized medical treatments, moving away from a “one-size-fits-all” paradigm.

While these innovations promise unprecedented functionalities for treating skeletal diseases, they also introduce new questions and challenges extending beyond the engineering limits. Advances in material selection and design techniques recently enable multifunctional properties, facilitated by cutting-edge developments in machine learning and 4D printing technologies. Such advancements could usher in a new era of hyper-personalized medical treatments that transcend mere physical compatibility, by incorporating real-time adaptability to physiological changes. The employment of AI in the design process could elevate this personalization further. For instance, future meta-implants may not only offer a precise fit within a patient's body but also actively respond to dynamic changes in their tissue environment, thereby redefining conventional concepts of medical devices and treatments.

However, the combination of AI and intricate materials science in healthcare brings forth substantial ethical and regulatory considerations. Issues related to long-term safety, data privacy, and the potential introduction of biases via machine learning could pose obstacles to clinical applications. Regulatory frameworks must evolve to accommodate these emerging technologies, balancing the imperative for innovation against concomitant risks to patients.

Emerging technologies such as multi-material 3D/and 4D printing not only offer tantalizing possibilities but also evoke new questions, including environmental sustainability concerns. As advancements in medical science are pursued, the ecological footprint of these burgeoning technologies must be critically assessed.

As the technological frontier of meta-implants continues to expand, interdisciplinary collaboration becomes increasingly vital. Material scientists, biomedical engineers, data scientists, and clinicians need to collaborate closely to fully realize the potential of meta-implants. Although the current trajectory of research and development appears promising, it necessitates ongoing vigilance concerning these wider implications.

## IX. CONCLUSION

Meta-biomaterials and meta-implants represent a revolutionary development in biomedical engineering. These engineered materials, with their unique microarchitectures, offer a blend of mechanical, physical, and biological properties, making them highly effective for treating complex skeletal diseases. The perspective presented in this paper focuses on diverse meta-implants, including hybrid, shape-morphing/shape-shifting, metallic clay, and deployable meta-implants, each showcasing unprecedented properties with various contributions to the field.

Hybrid meta-implants, combining various auxetic vs non-auxetic compartments, provide improved mechanical performance and longer service lives as compared to traditional implants. Shape-morphing meta-implants utilize (non-auxetic) meta-biomaterials and self-folding techniques for cost-effective shape customization, potentially also offering better stability and stimulation. Metallic clay meta-implants offer a cost-effective alternative to patient-specific 3D printed implants, while being capable of transitioning between shape-morphing and locked-shape states. Deployable meta-implants, inspired by origami and kirigami principles, demonstrate promising applications through optimized deployment force, mechanical properties, and surface nanopatterning.

Future developments of meta-implants should focus on the exploration of new avenues for incorporating multi-functionalities in meta-biomaterials and their application across diverse biomedical fields. Integration of rational micro-architectural design principles, machine learning, and AI tools holds promise for the development of multi-functional meta-implants with unique, multi-functional properties. Material selection, surface bio-functionalization, drug delivery mechanisms, and emerging 4D printing techniques represent critical areas for further advancement and refinement of meta-implant design.

To maximize the transformative impact of meta-implants in biomedical engineering, interdisciplinary collaboration and addressing certain technological bottlenecks are essential. Ongoing research and development to enhance multi-functional capabilities and design methodologies of these materials are vital for their effective use in complex skeletal diseases. Harnessing the unique properties of meta-biomaterials, meta-implants have the potential to revolutionize the currently existing treatment strategies and significantly improve patient outcomes in the field of orthopedics and beyond.

## Data Availability

Data sharing is not applicable to this article as no new data were created or analyzed in this study.
